# Self‐Curable Synaptic Ferroelectric FET Arrays for Neuromorphic Convolutional Neural Network

**DOI:** 10.1002/advs.202207661

**Published:** 2023-03-27

**Authors:** Wonjun Shin, Jiyong Im, Ryun‐Han Koo, Jaehyeon Kim, Ki‐Ryun Kwon, Dongseok Kwon, Jae‐Joon Kim, Jong‐Ho Lee, Daewoong Kwon

**Affiliations:** ^1^ Department of Electrical and Computer Engineering Inter‐University Semiconductor Research Center Seoul National University Seoul 08826 Republic of Korea; ^2^ Department of Electronic Engineering Hanyang University Seoul 04763 South Korea; ^3^ Present address: Ministry of Science and ICT Sejong 30121 Republic of Korea

**Keywords:** in‐memory‐computing, low‐frequency noise (LFN), selective detection, tungsten oxide

## Abstract

With the recently increasing prevalence of deep learning, both academia and industry exhibit substantial interest in neuromorphic computing, which mimics the functional and structural features of the human brain. To realize neuromorphic computing, an energy‐efficient and reliable artificial synapse must be developed. In this study, the synaptic ferroelectric field‐effect‐transistor (FeFET) array is fabricated as a component of a neuromorphic convolutional neural network. Beyond the single transistor level, the long‐term potentiation and depression of synaptic weights are achieved at the array level, and a successful program‐inhibiting operation is demonstrated in the synaptic array, achieving a learning accuracy of 79.84% on the Canadian Institute for Advanced Research (CIFAR)‐10 dataset. Furthermore, an efficient self‐curing method is proposed to improve the endurance of the FeFET array by tenfold, utilizing the punch‐through current inherent to the device. Low‐frequency noise spectroscopy is employed to quantitatively evaluate the curing efficiency of the proposed self‐curing method. The results of this study provide a method to fabricate and operate reliable synaptic FeFET arrays, thereby paving the way for further development of ferroelectric‐based neuromorphic computing.

## Graphical abstract

The primary challenge that ferroelectric field‐effect transistors face is their vulnerability to the repeated program/erase cycle. To solve this issue, an efficient self‐curing method is presented. The proposed method successfully recovers synaptic fatigue damage, enhancing learning accuracy in the convolutional neural network.

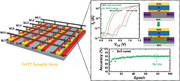

## 1 Introduction

Recent advances have allowed deep learning algorithms to outperform conventional machine learning techniques in a wide range of applications, including image classification and speech recognition.^[^ ^1^, ^2^, ^3^ ^]^ Specifically, the convolutional neural network (CNN) architecture which extracts useful features from image using kernels as filters, does not suffer from the burden of input data engineering, resulting in higher learning accuracy.^[^ ^4^, ^5^ ^]^ However, CNNs based on conventional digital computing systems have intrinsic drawbacks owing to the physical separation between processing and memory units.^[^ ^6^, ^7^ ^]^ The resulting limited data transfer rate (i.e., von Neumann bottleneck) incurs significant energy consumption and latency, which represent fundamental obstacles in the development of artificial intelligence.

Inspired by the human brain, neuromorphic systems have been extensively studied to overcome the von Neumann bottleneck.^[^ ^8^, ^9^, ^10^ ^]^ The neuromorphic hardware mimics the functional and structural features of a biological neural system with massive parallelism of neurons and synapses. Spikes from presynaptic neurons are transferred to postsynaptic neurons, with the synaptic weight determining the relative strength of this transfer. The plasticity of synapses whose weights can be modified during learning and maintained over time enables a parallel function of learning and memory. Similarly, memory and processing units are co‐located in the neuromorphic hardware, allowing information storage and processing to occur simultaneously.^[^ ^8^ ^]^ The synaptic device mimics the function of a biological synapse by taking voltage input from the presynaptic neurons and emitting the current output to the postsynaptic neurons. The conductance stored in the synaptic device controls the extent to which the input voltage is amplified into the output current, and the synaptic weight is updated according to the learning principles. Throughout this process, vector‐matrix‐multiplication (VMM) enables parallel data processing in the synaptic array, thereby drastically reducing the power consumption of the neuromorphic hardware.^[^ ^9^ ^]^

As the neuromorphic system is primarily composed of synaptic devices, a significant amount of research effort has been put into the development of such devices, most of which are based on two‐terminal memristors.^[^ ^11^, ^12^, ^13^ ^]^ Although the simple structure of two‐terminal memristors enables their integration into the system with a high density, the crossbar array structure exhibits crosstalk and sneak current path problems.^[^ ^14^, ^15^ ^]^ A sneak path carries unwanted current during the reading process, leading to extra energy consumption from unselected cells and degrading the update‐read accuracy. To prevent the sneak path current, an additional selector device is required, which inevitably increases the size of the array and nullifies the primary benefit of memristors.^[^ ^16^ ^]^

To circumvent the constraints of existing neuromorphic systems based on two‐terminal memristors, three‐terminal synaptic transistors have been developed.^[^ ^17^, ^18^, ^19^, ^20^ ^]^ These individually programmable transistors eliminate crosstalk and sneak path current between adjacent devices, enabling the weight update process to be conducted in a selective and parallel manner. In comparison to other types of three‐terminal synaptic transistors such as charge trap‐based synapses,^[^ ^17^, ^18^ ^]^ hafnium‐oxide‐based ferroelectric field‐effect transistors (FeFETs) offer the benefits of low program bias, quick switching speed, high scalability, and complementary‐metal‐oxide‐semiconductor compatibility.^[^ ^19^, ^20^ ^]^ However, two significant challenges must be resolved to successfully integrate FeFET into neuromorphic computing as a synaptic device. First, as the synaptic weight is updated iteratively during in situ training, the synaptic device should handle multiple program operations. However, existing FeFETs have poor endurance performance, with values of 10^4^–10^6^ or lower.^[^ ^21^, ^22^ ^]^ Furthermore, prior studies were largely restricted to single‐device‐level inquiries.^[^ ^23^, ^24^ ^]^ It is critical, however, to examine how the characteristics of a single synaptic device are reflected throughout an entire neuromorphic system. Considering these two aspects, it is necessary to establish a method that enhances the durability of FeFETs and enables quantitative device‐to‐system‐level characterization.

The present study addresses the aforementioned issues by improving the endurance of hafnium zirconium oxide (HZO) FeFETs via a self‐curing method and demonstrating the device‐to‐system‐level implementation of a synaptic FeFET array in a neuromorphic system. First, synaptic behaviors of FeFETs are demonstrated using the partial polarization of HZO. The weight of a synaptic FeFET can be selectively updated using program‐inhibit operations, and each FeFET implements kernel weights for convolution operations. The constructed synaptic FeFET array yields an image learning accuracy of 79.84% on the Canadian Institute for Advanced Research (CIFAR)‐10 dataset. The degradation of learning accuracy due to repetitive weight updating can be fully recovered using the proposed self‐curing method based on punch‐through current (*I*_punch_). Consequently, the results of this study pave a promising avenue to enable reliable synaptic FeFETs to be integrated into neuromorphic computing.

## 2 Results and Discussion

### 2.1 System Architecture

In machine learning algorithms for image recognition, feature extraction from objects is a critical process that reduces the original data's dimensionality into a new space. Inspired by the human vision system, recently developed algorithms do not evaluate entire images pixel‐by‐pixel for recognition; instead, they compare only the extracted features with the learned memory, thereby reducing the burden of work and energy consumption. A CNN is a representative learning algorithm that performs feature extraction with high efficiency.^[^ ^4^ ^]^ Inspired by the human visual cortex, a CNN is comprised of a number of convolutional and pooling layers that are used to extract invariant features from input patterns. Recent studies have reported that the CNN architecture yields outstanding results in terms of learning accuracy and energy efficiency compared to other types of learning algorithms. However, when implementing a CNN using conventional von Neumann architecture, the limited data transfer rate between the processor and memory unit increases the energy consumption and process latency.

**Figure 1:**
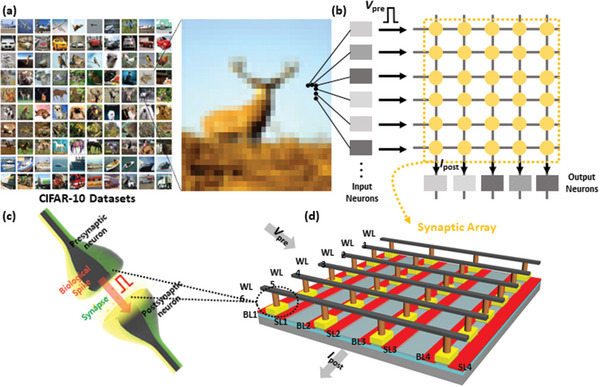
a) CIFAR‐10 dataset. b) Schematic of feature extraction in the convolutional neural network. The input neuron is connected to a pixel in the image and emits *V*_pre_, whose magnitude is defined by the pixel intensity. Then, the *V*_pre_s, which triggers synaptic devices comprising the convolutional layer and corresponding channel conductance, are cumulatively added to an output neuron. When the accumulated postsynaptic current level exceeds a given threshold value, one output neuron fires a *V*_post_. c) Schematic of biological neuron and synapse. d) Schematic of synaptic ferroelectric field‐effect‐transistor array composed of word lines (WLs), bit lines (BLs), and source lines (SLs).

The neuromorphic system can significantly improve the efficiency of a CNN by adopting VMM, which enables parallel data processing in the synaptic array. The learning principle of neuromorphic‐based CNNs can be explained as follows: The intensity of the pixels determines the magnitude of the presynaptic spikes (*V*_pre_) emitted by the input neuron associated with a pixel. The *V*_pre_s, which triggers synaptic devices and channel conductance of the convolutional layer, is then added to an output neuron. If the accumulated postsynaptic current level exceeds a certain threshold, a postsynaptic spike (*V*_post_) is fired. According to the correlation between the pre‐ and postsynaptic spikes, the synaptic weight is adjusted to a certain analog state. The overall process is illustrated in **Figure** **1**a,b. In this study, the three‐terminal HZO FeFET array was employed to emulate synaptic behavior, as shown in Figure 1c,d. During in situ training, the synaptic weights must be updated accurately and selectively over multiple iterations. Two major requirements must therefore be satisfied to successfully implement a FeFET as a synaptic device: 1) The multilevel synaptic weight should be selectively updated to the targeted synaptic device (program) without interfering with the untargeted device (inhibition). 2) High durability of the synaptic device should be guaranteed for multiple program/erase operations. However, most prior studies demonstrated the multilevel synaptic weight in a single device without considering the program‐inhibit in the synaptic array. In addition, conventional FeFETs suffer from poor durability, and their endurance must be improved.

### 2.2 Demonstration of Synaptic FeFETs From a Single Transistor to the Array Level

We first demonstrate that the fabricated FeFET array successfully mimics the function of a biological synapse. A remnant polarization of ferroelectric material can be tuned by the electrical bias, and partial polarization can be achieved by applying different biases.^[^ ^25^, ^26^, ^27^ ^]^ In this study, a multilevel conductance is realized by utilizing the partial polarization of HZO. The ferroelectricity of the HZO film is investigated using metal /ferroelectric /insulator /semiconductor (MFIS) capacitance having the same structure as the fabricated FeFET. The positive‐up‐negative‐down (PUND) approach is used to measure the polarization characteristics of the HZO film. The corresponding measured current–voltage curve of the HZO is shown in Figure S1a, Supporting Information.

Likewise, Figure S1b, Supporting Information, depicts the polarization versus voltage curve with an increase in voltage sweep range. The MFIS capacitance exhibits an increase in both positive and negative remnant polarization with an increase in bias sweep range, confirming partial polarization in the HZO film. Note that the HZO film exhibits a 27.5 µC cm^−2^ and _−_26.7 µC cm^−2^ in the sweep range from −6.5 V to + 6.5 V. Thus, partial polarization successfully emulates synaptic behavior.

The effects of polarization switching on memory behaviors in a single FeFET are subsequently investigated. The fabrication process of the single FeFET is illustrated in Figure S2, Supporting Information. The FeFET's threshold voltage (*V*_th_) can be modulated by applying bias to the gate, thereby changing the HZO polarization. When the program pulse is applied to the gate, the HZO is polarized to a Si direction, and the electron concentration in the FET channel subsequently increases, thereby decreasing *V*_th_. Contrarily, when the erase pulse is applied to the gate, *V*_th_ increases because polarization is induced to the TiN direction. Figure S3a,b, Supporting Information, illustrates the erase and program characteristics of the single FeFET, respectively. Note that the FeFET's switching speed far exceeds that of the previously reported ferroelectric thin‐film transistor in the synaptic array (10 ms).^[^ ^28^ ^]^ In addition, the retention characteristics of the single FeFET are investigated in both program and erase states. As shown in Figure S4a, Supporting Information, the FeFET exhibits excellent retention characteristics. Figure S4b, Supporting Information, shows the corresponding 2*P*_r_ retention characteristics of the FeFET. Note that retention characteristics were measured at 27 °C.

**Figure 2:**
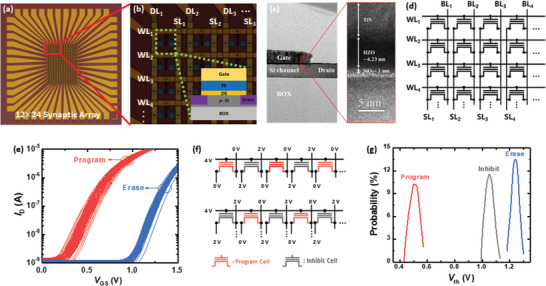
a) Top optic image of fabricated synaptic ferroelectric field‐effect‐transistor (FeFET) array with dimensions of 12 × 24. b) Enlarged optic image of FeFET in Figure 1a. The source line (SL) and bit line (BL) are in parallel, with the word line (WL) being perpendicular to both. The inset represents a gate stack schematic of the unit FeFETs comprising the array. c) TEM image of a unit FeFET. d) Schematic of synaptic FeFET array. e) *V*_th_ distribution of 25 synaptic FeFETs following program and erase operations. f) Program‐inhibit bias scheme applied to the array. g) *I*_D_s (unit of nA) of FeFETs in synaptic array following parallel program/inhibit operations.

Accordingly, we investigate the synaptic characteristics of the FeFET array. The CMOS‐compatible synaptic FeFET array is fabricated with dimensions of 12 × 24 (**Figure** **2**a). Within the array, the gate, source, and drain electrodes of the FeFETs can be approached via word lines (WLs), source lines (SLs), and bit lines (BLs), respectively (Figure 2b). The SL and BL are in parallel, with the WL being formed perpendicular to both. The inset represents a schematic of the unit FeFETs comprising the array. The TEM image of the unit FeFET is illustrated in Figure 2c, and a schematic of the fabricated synaptic array is presented in Figure 2d.

The switching properties of FeFETs are investigated by applying program (+ 4 V, 10 µs) and erase (_−_4 V, 100 µs) pulses. Figure 2e illustrates the transfer characteristic (*I*_D_–*V*_GS_) distribution of 25 synaptic FeFETs in the array following the program and erase operations. These results demonstrate that the *V*_th_ of each FeFET can be successfully tuned. Figure S5a, Supporting Information, shows the corresponding *V*_th_ distribution after program and erase operations. In the synaptic array, it is critical to selectively program and erase each cell without affecting the adjunct cells,^[^ ^28^, ^29^ ^]^ which necessitates the inhibit operations. Five consecutive devices are selected to test the program‐inhibit operation. To program each selected device, the program pulse (+ 4 V, 10 µs) is applied to the WL, while the inhibit pulse (+ 2 V, 10 µs) is applied to the BL of the unselected device. The inhibit pulse reduces the voltage difference between the WL and channel of the unselected device, thereby preventing polarization switching. Two cases of program‐inhibit operations are tested in the synaptic array. The bias condition for each case is noted in Figure 2f. Figure 2g represents the probability of *V*_th_ distribution in the synaptic array following parallel program/erase‐inhibit operations. The selected device exhibited an ≈0.6 V larger *V*_th_ than the inhibited device in both cases, demonstrating excellent program‐inhibit performance. Figure S5b, Supporting Information, shows the corresponding conductance mapping of the synaptic FeFETs, exhibiting the program‐inhibit operation.

**Figure 3:**
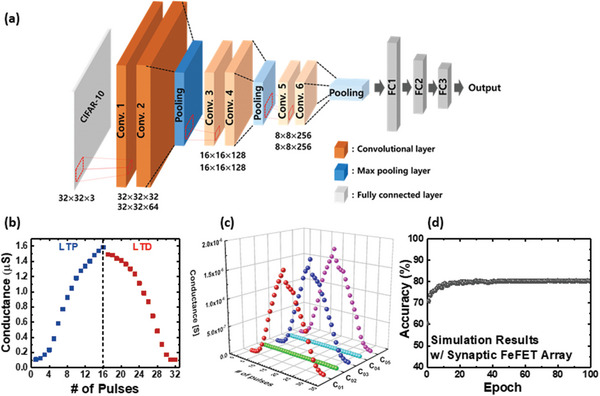
a) Nine‐layer visual geometry group network schematic. b) Long‐term potentiation/depression (LTP/LTD) characteristics of selected and inhibited ferroelectric field‐effect‐transistors (FeFETs) in the array. c) LTP/LTD characteristics of selected and inhibited FeFETs in the array. The program‐inhibit operation was successfully achieved at the array level. d) FeFET learning accuracy versus epoch.

The applicability of the fabricated synaptic array to neuromorphic computing is examined by testing the VMM. The VMM algorithm, which is based on Ohm's and Kirchhoff's laws, can be used to achieve parallel data processing in neuromorphic computing. As synaptic devices can precisely adjust conductance states via analog conductance modulation, they are essential to ensure the accuracy of VMM. To evaluate the synaptic array's performance, a nine‐layer visual geometry group (VGG‐9) network^[^ ^30^ ^]^ is simulated using the  CIFAR‐10 dataset. This VGG‐9 network comprises six convolutional layers, three max‐pooling layers, and two fully‐linked layers, wherein one max‐pooling layer is employed for each pair of convolutional layers. All input CIFAR‐10 images are 32 × 32 × 3 pixels in size, and kernels with three weights are employed for the convolutional layers. When CIFAR‐10 pictures are processed via the first and second convolutional layers, the feature maps are 32 × 32 × 32 and 32 × 32 × 64 pixels in size, respectively. Following the third and fourth convolutional layer operations, 16 × 16 × 128 feature maps are generated. Subsequently, the fifth and sixth convolutional layer procedures are used to acquire feature maps with a size of 8 × 8 × 256. All feature maps are connected to the fully connected layers. **Figure** **3**a illustrates a schematic of the VGG‐9 network used in this study.

As the synaptic weight is a reflection of synaptic connectivity between presynaptic and postsynaptic neurons, it must be selectively updated during learning. To achieve this, an incremental pulse scheme is used for the linear weight update, which ensures a high learning accuracy.^[^ ^31^, ^32^ ^]^ The pulse amplitudes of potentiation and depression are increased from 2.3 to 3.95 V in a 0.11 V step, and from −2.2 to −4.1 V in a −0.14 V step, respectively. Note that the pulse width is fixed to 10 µs. Figure 3b shows excellent long‐term potentiation/depression (LTP/LTD) characteristics of a single FeFET in the array. Beyond the single transistor level, the LTP/LTD characteristics must be selectively realized for accurate learning in the synaptic array. To inhibit unselected synapses during the weight update, the inhibit pulse (+ 2 V, 10 µs) is applied to each unselected device. Figure 3c illustrates the LTP/LTD characteristics of selected (C_01_, C_03_, and C_05_) and inhibited (C_02_ and C_04_) FeFETs. Note that the locations of all five devices are shown in Figure S6, Supporting Information. It is clearly observed that the synaptic weights of the selected FeFETs are selectively updated during learning, while those of the unselected FeFETs are inhibited.

Based on the synaptic characteristics of FeFETs—including LTP/LTD, selective weight updating, and *V*_th_ variation—the VGG‐9 network is simulated using the CIFAR‐10 dataset. Figure 3d represents a plot of the array‘s learning accuracy versus epoch. The system exhibits an excellent overall learning accuracy of 79.21% on the CIFAR‐10 dataset.

### 2.3 Self‐Curable Synaptic FeFET

**Figure 4:**
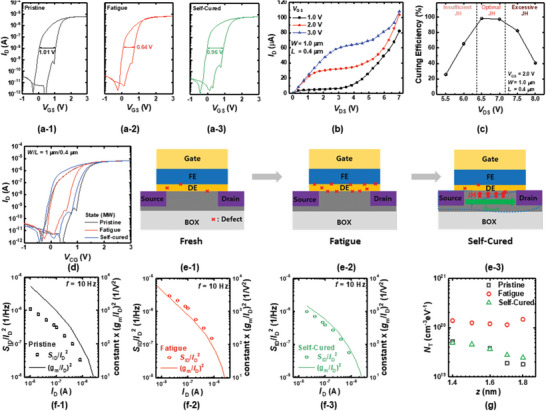
Transfer characteristics (*I*_D_–*V*_GS_) of a‐1) pristine, a‐2) fatigue, and a‐3) self‐cured FeFETs. b) Output characteristics (*I*_D_–*V*_DS_) of the FeFET measured at different *V*_GS_ values. c) Curing‐efficiency of the *I*_punch_‐based self‐curing versus *V*_DS_. d) Combined *I*_D_–*V*_GS_ of all ferroelectric field‐effect transistors (FeFETs) in (a). Schematic defect generation and curing mechanism of (c‐1) pristine, (c‐2) fatigue, and (c‐3) self‐cured FeFETs. Normalized drain current power spectral density (*S*_ID_/*I*_D_^2^) and (*g*_m_/*I*_D_)^2^ of (c‐1) pristine, (c‐2) fatigue, and (c‐3) self‐cured FeFETs versus *I*_D_. e) Volume trap density (*N*_T_) along the vertical distance (*z*) from the channel‐gate oxide interface to gates of pristine, fatigue, and self‐cured FeFETs.

Although the fabricated synaptic FeFET array exhibits excellent performance with a CNN, the degradation of LTP/LTD characteristics caused by repeated weight updates during in situ learning can severely degrade the learning accuracy, in a phenomenon known as synaptic fatigue. **Figure** **4**a‐1 illustrates the DC double sweep transfer characteristics (*I*_D_–*V*_GS_) of a single FeFET in the pristine state, exhibiting a memory window of 1.01 V. However, this memory window is decreased to 0.64 V following 10^5^ iterations of the potentiation/depression cycle, as shown in Figure 4a‐2. The corresponding degradation of the 2*P*_r_ of the device is shown in Figure S7, Supporting Information. This degradation effect originates from cumulative defects at the gate oxide,^[^ ^33^, ^34^, ^35^ ^]^ wherein a large electric field is applied to the FE/DE interface by the polarization of HZO, causing severe charge trapping and defect generation. Accordingly, the synaptic characteristics of the FeFET are distorted as a result. Synaptic fatigue is not unique to the devices used in this study but inherent to the structural specificity of FeFETs. Consequently, most prior studies report an endurance between 10^4^ and 10^6^ or even lower.^[^ ^21^ ^]^ It is therefore crucial to improve the endurance of FeFETs irrespective of structure.

A self‐curing method using electrothermal annealing (ETA) may represent a means to improve the endurance of FeFETs to synaptic fatigue. ETA utilizes the Joule heat (JH) generated during transistor functions, including punch‐through, gate‐induced drain leakage (GIDL), and body‐bias curing.^[^ ^36^, ^37^, ^38^, ^39^ ^]^ As self‐curing methods utilize the internally generated JH of transistors, they do not require bulky external equipment such as a furnace for global annealing. Most importantly, because ETA can be applied even after packaging, its curing effects can be applied selectively to a damaged device even in the process of in situ training. We therefore propose a self‐curing method based on *I*_punch_. As defects are generated throughout the entire channel along the lateral dimension of gate oxide during weight updates, it is crucial to generate the JH uniformly along the conductive channel. In this regard, the GIDL and body‐bias‐induced current are not appropriate for the curing as they locally generate the JH at the drain–channel junction.

Figure 4a‐3 shows the *I*_D_–*V*_GS_ of the FeFET after the self‐curing is applied based on *I*_punch_. Note that *V*_GS_ = 2.0 V and *V*_DS_ = 6.5 V are applied for 1 ms to induce punch‐through. Figure 4b shows the output characteristics (*I*_D_–*V*_DS_) of the FeFET measured at different *V*_GS_ values. A rapid increase in *I*_D_ is observed with an increase in *V*_DS_ due to the punch‐through leakage. The *V*_GS_ of 1.0 V is too small to induce sufficient JH due to the small *I*_punch_. The *V*_GS_ of 3.0 V is unnecessary, considering the power consumption. Therefore, the *V*_GS_ of 2.0 V is selected. Figure 4c shows the curing efficiency of the *I*_punch_‐based self‐curing measured at different *V*_DS_ values. Note that the curing efficiency is defined as follows:

(1) $$\mathrm{Curing}\;\mathrm{Efficiency}=\left(1-\frac{{\mathrm{MW}}_{P}-{\mathrm{MW}}_{\mathrm{SC}}}{{\mathrm{MW}}_{P}}\right)\times 100\left(\%\right)$$

where MW_P_ and MW_SC_ denote the memory window of the pristine and self‐cured FeFETs. The curing efficiency is the largest at *V*_DS_ = 6.5 V, which generates the efficient JH to repair the damage caused by the P/E cycling. An excessive *V*_DS_ induces additional damage to the device, resulting in a decrease in curing efficiency. The JH caused by *I*_punch_ is effectively transferred to defects inside DE and FE due to the low thermal conductivity of the SiO_2_ comprising the SOI wafer (≈1 W mK^−1^). It is clearly observed that the memory window of the FeFET almost fully recovers to its original value (0.96 V). Figure 4d illustrates the *I*_D_–*V*_GS_ of pristine, fatigued, and self‐cured FeFETs, demonstrating excellent curing efficiency. The curing mechanism is schematically summarized in Figure 4e. Figure S8, Supporting Information, shows pristine, fatigued, and self‐cured FeFETs under varying channel dimensions. Note that curing efficiency decreases with an increase in channel length, while exhibiting a weak dependence on channel width. This is because a larger bias is required to induce the punch‐through at a longer channel length. Here it is noteworthy to mention that the power consumption for self‐curing is decreased with the scaling down of the channel length because the lower *V*_DS_ is required to induce the punch‐through. Therefore, the proposed self‐curing method is more energy‐efficient in highly scaled‐down FeFETs, which is beneficial for low‐power operation.

To quantitatively evaluate the proposed method's curing efficiency, LFN spectroscopy was adopted. LFN spectroscopy is an established diagnostic tool to evaluate the reliability of semiconductor materials and devices, including FeFETs.^[^ ^40^, ^41^, ^42^, ^43^, ^44^ ^]^ Unlike other electrical measurements, such as current–voltage characterization and the charge pumping method, LFN spectroscopy measures not only the defects at the gate oxide‐channel interface but also the bulk defects. In FETs, LFN is generated from the carrier trapping/de‐trapping processes to/from defects in the gate oxide, and the defect density can be quantitatively characterized by measuring the power spectral density (PSD) of all devices. This behavior is reflected by the carrier number fluctuation (CNF) model, which is described as^[^ ^45^, ^46^ ^]^

(2) $$\frac{S_{\mathrm{ID}}}{I_{D}^{2}}={\left(\frac{g_{m}}{I_{D}}\right)}^{2}\frac{q^{2}k_{B}TN_{T}\lambda }{WLC_{\mathrm{ox}}^{2}f}$$

where *g*_m_ is transconductance, *q* is the electron charge, *k*_B_ is the Boltzmann constant, *N*_T_ is the volume trap density, *λ* is the tunneling attenuation coefficient, *C*_ox_ is the gate oxide capacitance per unit area, and *f* is the frequency. Figure S9, Supporting Information, shows the normalized *I*_D_ PSD (*S*_ID_/*I*_D_^2^) of pristine, fatigued, and self‐cured FeFETs measured at *I*_D_ = 200 nA. In all cases, the FeFETs exhibit 1/*f* noise behavior, which increases following *P*/*E* cycle‐induced stress and decreases to its original value after self‐curing is applied. To verify whether the 1/*f* noise of the FeFETs originates from the CNF, the *S*_ID_/*I*_D_^2^ sampled at 10 Hz and (*g*_m_/*I*_D_)^2^ are plotted as a function of *I*_D_, as shown in Figure 4f. In all cases, the *S*_ID_/*I*_D_^2^ and (*g*_m_/*I*_D_)^2^ exhibit equivalent behavior with an increase in *I*_D_, demonstrating that the 1/*f* noise of the FeFETs originates from the CNF.

As the 1/*f* noise stems from the CNF, the trap density (*N*_T_) along the vertical direction inside the gate oxide can be extracted from the PSD according to the following equation:^[^ ^47^, ^48^ ^]^

(3) $$z=\lambda \ln\left(\frac{1}{2\pi f\tau_{0}}\right)$$

where *z* is the vertical distance from the channel/gate oxide interface, and *τ*_0_ is the time required for tunneling into a trap state at the interface (*z* = 0). Figure 4g plots the *N*_T_ in pristine, fatigued, and self‐cured FeFETs versus vertical depth along the gate oxide. It is clearly shown that the increased *N*_T_ following synaptic fatigue is reduced to its original value after the self‐curing is applied, quantitatively demonstrating the proposed method's excellent curing efficiency.

### 2.4 Application of Self‐Curing Method to Neuromorphic Computing

**Figure 5:**
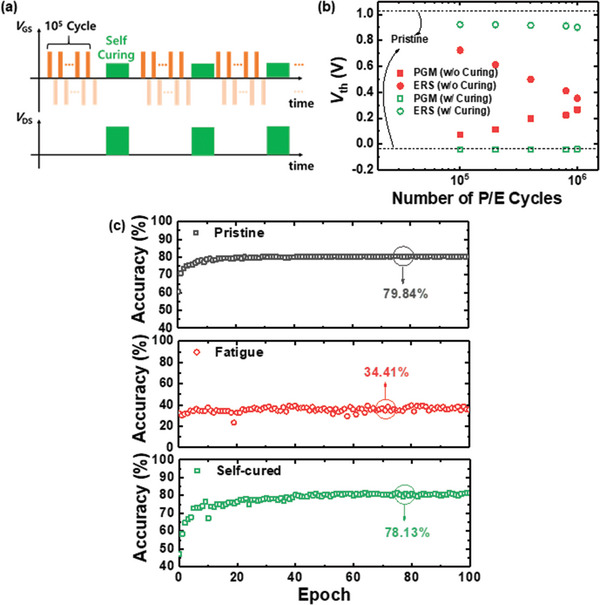
a) Bias scheme for enhancement of endurance against synaptic fatigue. b) Threshold voltages (*V*_th_) of ferroelectric field‐effect transistors (FeFETs) at the program and erase states as functions of the number of program/erase (P/E) cycles. c) Learning accuracy of pristine, fatigued, and self‐cured FeFETs versus epoch.

The proposed self‐curing method based on ETA significantly improves the performance of synaptic FeFETs. Accordingly, we designed an efficient bias scheme for the endurance enhancement of said devices. As the number of weight updates during in situ learning exceeds a certain threshold, the FeFET is permanently damaged due to a collapse in the memory window. In the fabricated FeFETs, this collapse is observed after 10^6^ program/erase (*P*/*E*) cycles. We also found that the device could not be recovered following the collapse even using ETA. Accordingly, we apply ETA to recover the damaged device prior to the collapse of the memory window. **Figure** **5**a illustrates the proposed bias scheme for self‐curing, wherein ETA is applied following 10^5^ *P*/*E* cycles. Figure 5b shows the *V*_th_ of a FeFET with and without an application of the self‐curing method. After 10^6^ *P*/*E* cycles, the gate oxide exhibits a significant increase in defects, impeding the ferroelectric switching; and thus; collapsing the memory window. As shown in Figure 4e, the bulk defects in HZO likewise increase with cycling, which cannot be fixed by the JH. It is therefore critical to recover the device prior to the trap being formed. When the self‐curing method is applied to the device every 10^5^ *P*/*E* cycles, the memory window is maintained even after the threshold of 10^6^ cycles. The endurance to synaptic fatigue can thus be improved tenfold via ETA self‐curing. In addition, retention characteristics of the FeFETs with and without the application of the self‐curing method are investigated. Figure S10, Supporting Information, shows the retention characteristics of the device after the 10^5^ times of P/E cycles with and without the repeated self‐curing method. A significant improvement in retention characteristics is observed by adopting the self‐curing method.

Figure 5c presents a plot of learning accuracy versus epoch of pristine (79.84%), fatigued (34.41%), and self‐cured FeFETs (78.13%). When a synaptic FeFET is fatigued, learning cannot proceed. However, when the self‐curing method is applied, it is confirmed that the learning capacity is fully recovered. These results demonstrate the promising applicability of the self‐curing method to synaptic FeFETs.

The results presented in this study provide a comprehensive investigation and demonstration of synaptic characteristics in FeFET arrays. Despite the fact that a substantial volume of research has previously been conducted on synaptic FeFETs, two fundamental issues still remain: 1) Most prior studies solely examined synaptic properties at a single device level. 2) Existing methods for enhancing the endurance of FeFETs to synaptic fatigue rely mostly on the optimization of the fabrication process, such as thermal annealing, which cannot be exploited during in situ learning. By demonstrating the applicability of the self‐curing method to the synaptic FeFET array, the present study represents a suitable solution to the aforementioned issues. Note that the self‐curing methods have been applied to various transistors, including FeFET.^[^ ^49^ ^]^ However, this study proposed the punch‐through current‐based self‐curing method in the synaptic FeFET array level for the first time. More importantly, the effects of self‐curing method on the synaptic behavior of the FeFETs and CNN have not been reported. The novelty of the proposed method is explained in Note S1, Supporting Information, in detail.

Here, we want to emphasize that the use of the proposed self‐curing method is not restricted to the FeFETs utilized in this study. As punch‐through can be induced in FETs with source and drain junctions, the proposed curing method can be applied to all FeFETs by optimizing bias conditions. Therefore, we believe that implementing the proposed self‐curing method will be a significant breakthrough in the ferroelectric‐based neuromorphic system by solving the most crucial obstacle FeFETs experience, which is limited cycling endurance.

## 3 Conclusion

We constructed a synaptic FeFET array based on HZO and demonstrated its applicability to neuromorphic computing. By utilizing the partial polarization of HZO film, the multilevel conductance of synaptic weights was achieved. The program‐inhibit operation was also successfully realized at the array level, selectively programming the targeted synapses. Based on the LTP/LTD characteristics, CNN performance was evaluated on the CIFAR‐10 dataset, wherein the fabricated synaptic FeFETs exhibited an excellent learning accuracy of 79.84%. Furthermore, a self‐curing method based on *I*_punch_ was employed to improve the endurance of FeFET to synaptic fatigue owing to program/erase cycles by a factor of 10. The proposed method's excellent curing efficiency was quantitatively evaluated using LFN spectroscopy. The results obtained in this study indicate the potential of synaptic FeFETs to be successfully adopted into neuromorphic computing.

## 4 Experimental Section

### Fabrication Process of FeFETs

The FeFETs were fabricated on a lowly‐doped *p*‐type silicon‐on‐insulator (SOI) wafer with a device silicon thickness of 100 nm (Figure S3a, Supporting Information). The wafer was cleaned using an SPM solution (H_2_SO_4_: H_2_O_2_ = 4: 1), SC‐1 solution (NH_4_OH: H_2_O_2_: H_2_O = 1: 1: 5), SC‐2 solution (HCl: H_2_O_2_: H_2_O = 1: 1: 5), and diluted HF solution (HF: H_2_O = 1: 100) after active patterning (Figure S3b, Supporting Information). Next, the dielectric (SiO_2_) and ferroelectric (HZO) layers were deposited via ALD (Figure S3c, Supporting Information). Note that the deposition cycles of HZO film comprise two cycles of HfO_2_ and one cycle of ZrO_2_. The cycles were repeated 23 times, and the HfO_2_ cycle was repeated two additional times to form a 6.2 nm HZO. A 1.0 nm layer of SiO_2_ and 6.2 nm layer of HZO were formed as the dielectric and ferroelectric layers, respectively. Subsequently, 100 nm TiN was sputtered (Figure S3d, Supporting Information) and patterned for a gate metal and hard mask for implantation (Figure S3e, Supporting Information). Phosphorus ions were implanted on the source/drain region with a dose of 10^15^ cm^−2^ and energy of 10 KeV (Figure S3f, Supporting Information). Post‐metal annealing was performed using RTA at 500 °C for 30 s in N_2_ ambient to crystallize HZO film and activate dopants. Last, high‐pressure annealing (HPA) was conducted to improve the ferroelectricity of FeFETs. HPA was maintained at 400 °C in the forming gas ambient conditions (H_2_: 4% and N_2_: 96%) for 30 m.

### Electrical Measurement

The ferroelectricity of FeFETs was investigated using a parameter analyzer (Keithley 4200‐SCS) and current–voltage module (4225‐PMU). The *P*–*V* curves were measured via the PUND method in conjunction with a time‐transient measurement using a triangular pulse with a 2.5 kHz frequency. A semiconductor parameter analyzer (B1500A), low‐noise current amplifier (SR570), and signal analyzer (35670A) were used to measure the PSD of the constructed FTJs. B1500A was used to supply voltage to the top gate. The output current was fed to SR570, which converted the current fluctuation into a voltage fluctuation. Last, 35670A transformed the SR570 dynamic signal into the PSD.

## Conflict of Interest

The authors declare no conflict of interest.

## Supporting information

Supporting Information

## Data Availability

The data that support the findings of this study are available in the Supporting Information of this article.
