# Supplementary material for: Self‐Curable Synaptic Ferroelectric FET Arrays for Neuromorphic Convolutional Neural Network
Source: Adv Sci (Weinh). 2023 Mar 27;10(15):2207661. doi: 10.1002/advs.202207661 (PMC10214256; doi:10.1002/advs.202207661)
Supplement: Supplementary file 1 — Supporting Information [file ADVS-10-2207661-s001.pdf]

# Supporting Information

**Self-curable synaptic ferroelectric FET arrays for neuromorphic convolutional neural network**

*Wonjun Shin, Jiyong Im, Ryun-Han Koo, Jaehyeon Kim, Dongseok Kwon, Jae-Joon Kim, Jong-Ho Lee*, and Daewoong Kwon*

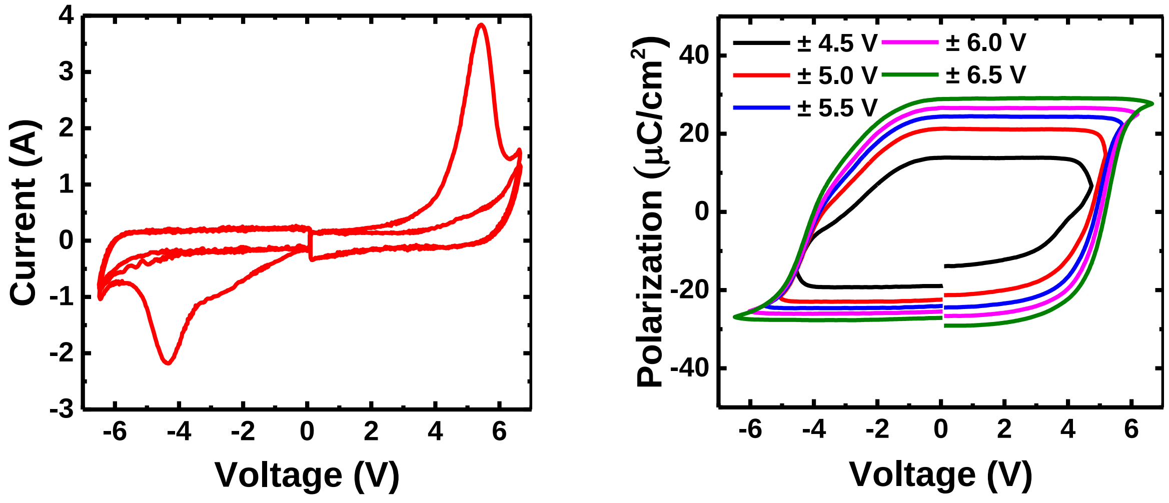


(a) (b)

Figure S1. Current-voltage curve of the HZO measured by PUND. (b) Polarization versus voltage curve with the increase in the voltage sweep range.

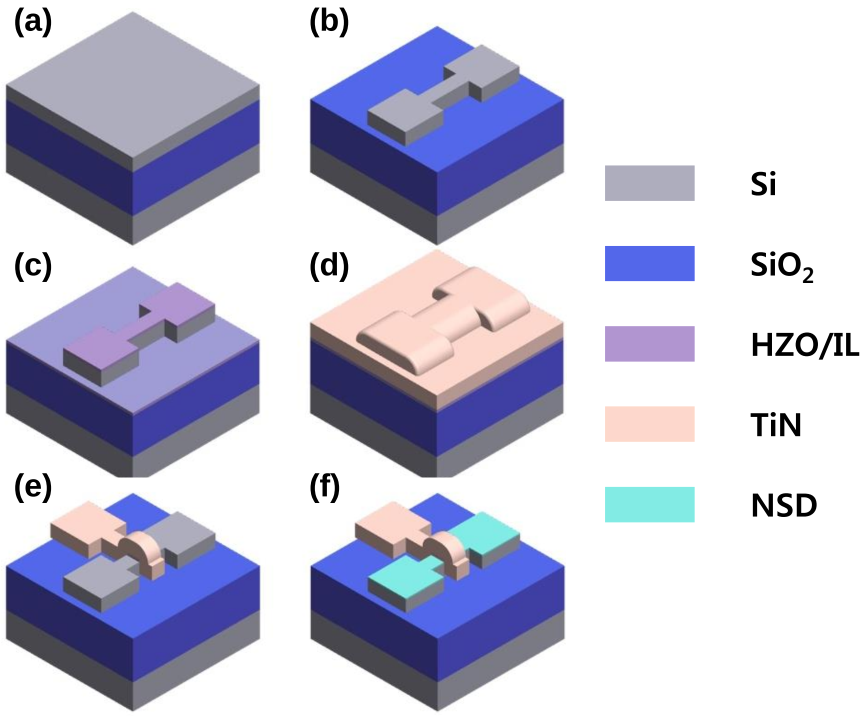


Figure S2. (a) The FeFETs were fabricated on a lowly doped *p*-type Silicon-On-Insulator (SOI) wafer where the device silicon thickness is 100 nm. (b) The wafer was cleaned using SPM solution ($H_2SO_4$: $H_2O_2$=4: 1), SC-1 solution ($NH_4OH$: $H_2O_2$: $H_2O$=1: 1: 5), SC-2 solution (HCl: $H_2O_2$: $H_2O$=1: 1: 5) and diluted HF solution (HF: $H_2O$=1: 100) after active patterning. (c) Next, dielectric layer ($SiO_2$) and ferroelectric layer (HZO) were deposited via ALD. Note that the deposition cycles of HZO film consisted with two cycles of $HfO_2$ and one cycle of $ZrO_2$. The cycles were repeated 23 times and $HfO_2$ cycle were repeated two more cycles to form a 6.2 nm HZO. A 1.0 nm of $SiO_2$ and a 6.2 nm of HZO are formed as dielectric and ferroelectric layers, respectively. Subsequently, (d) 100 nm TiN was sputtered and (e) patterned for a gate metal and a hard mask for implantation. (f) Phosphorous ions were implanted on the source/drain region with the dose of $10^{15}$ $cm^{-2}$ and the energy of 10 KeV. Post-metal annealing was performed using RTA at 500 ℃ for 30 s in $N_2$ ambient to crystallize HZO film and activate dopants. Finally, the high-pressure annealing (HPA) is conducted to improve the ferroelectricity of the FeFETs. The HPA is conducted at 400 $^{o}$C in the forming gas ambient conditions ($H_2$: 4% and $N_2$: 96%) for 30 m.

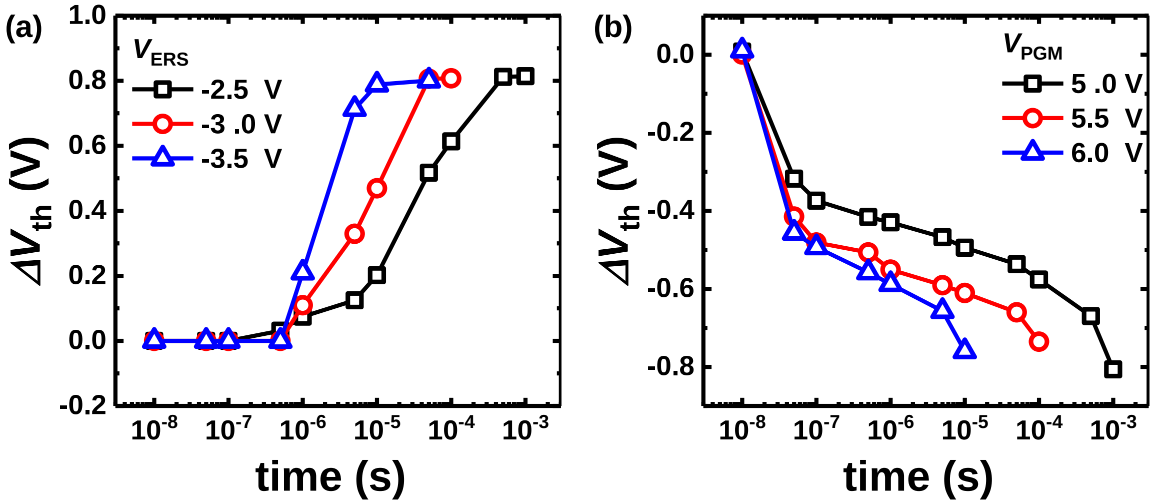


Figure S3. $\Delta V_{th}$ versus (a) erase pulse time and (b) program pulse time at different voltage magnitudes. The switching characteristics are measured at the single FeFET.

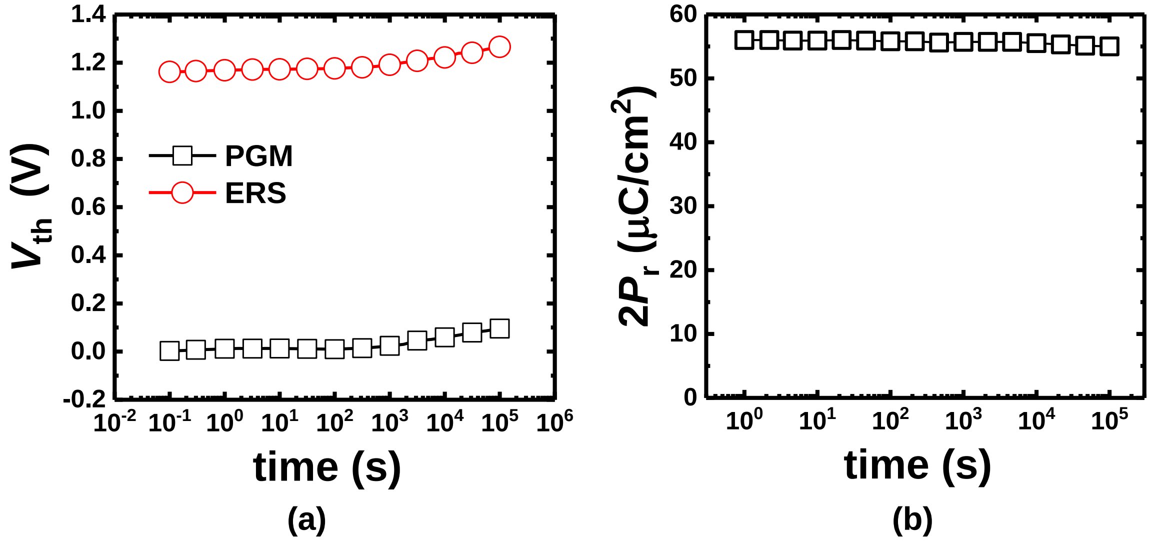


Figure S4. (a) Retention characteristics of the single FeFET. The retention characteristics are measured at 27 °C. (b) $2P_r$ value versus retention time of the FeFET.

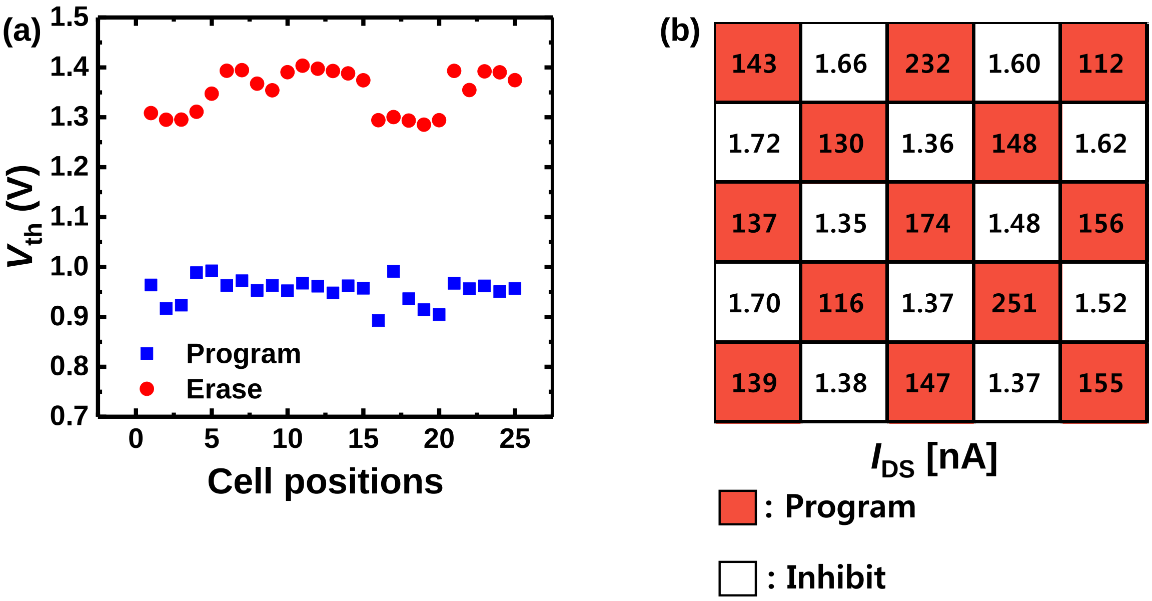


Figure S5. (a) $V_{th}$ distribution of the FeFETs after program and erase operations. (b) Conductance mapping of the synaptic FeFETs, exhibiting the program-inhibit operation.

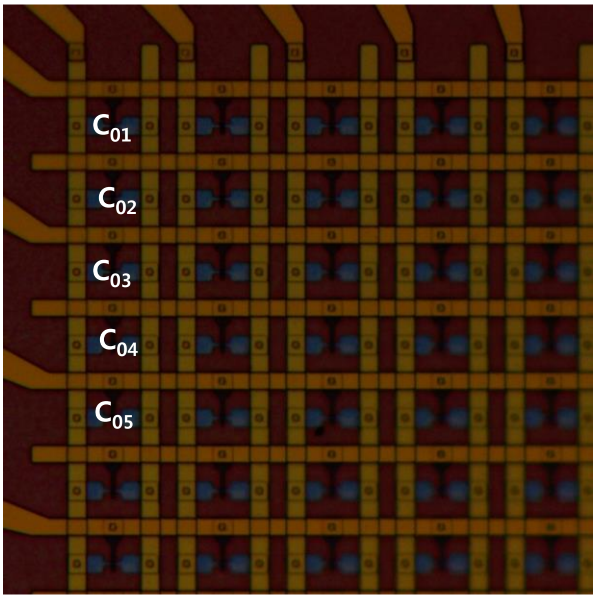


Figure S6. Location of $C_{01}$, $C_{02}$, $C_{03}$, $C_{04}$, and $C_{05}$ in the FeFET array.

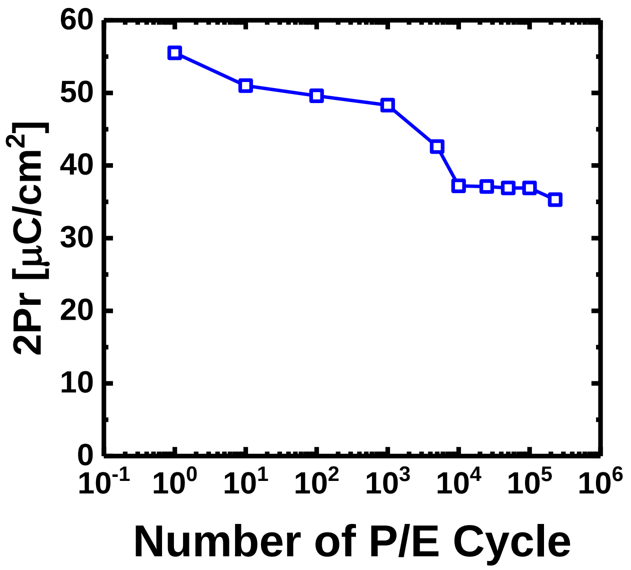


Figure S7. 2$P_r$ value versus the number of P/E cycle of the FeFETs.

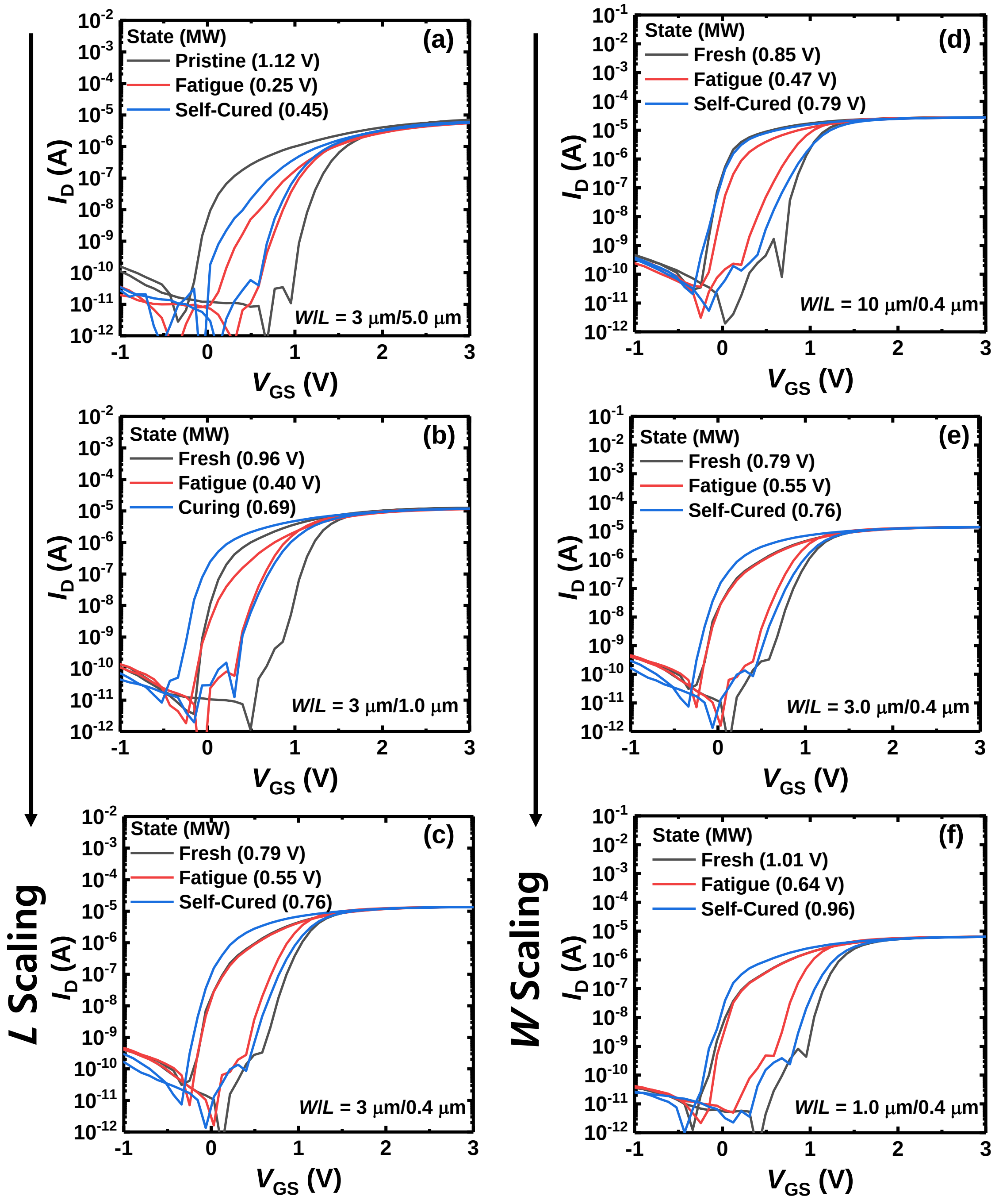


Figure S8. (a)-(c) Transfer characteristics ($I_D$-$V_{GS}$) of the FeFETs with respect to change in $L$ ((a): $W/L$ = 3.0 μm/5.0 μm, (b) $W/L$ = 3.0 μm/1.0 μm, and (c) $W/L$ = 3 μm/0.4 μm). (d)-(e) $I_D$-$V_{GS}$ of the FeFETs with respect to change in $W$ ((d): $W/L$ = 10 μm/0.4 μm, (e) $W/L$ = 3.0 μm/0.4 μm, and (f) $W/L$ = 1.0 μm/0.4 μm). The proposed self-curing method exhibit an excellent curing efficiency.

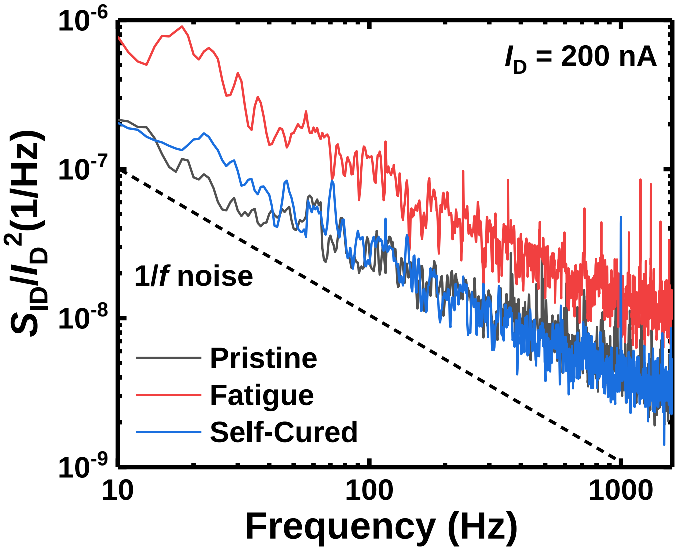


Figure S9. Normalized $I_D$ power spectral density ($S_{ID}/I_D^2$) of the pristine, fatigued, and self-cured FeFETs measured at $I_D$ = 200 nA.

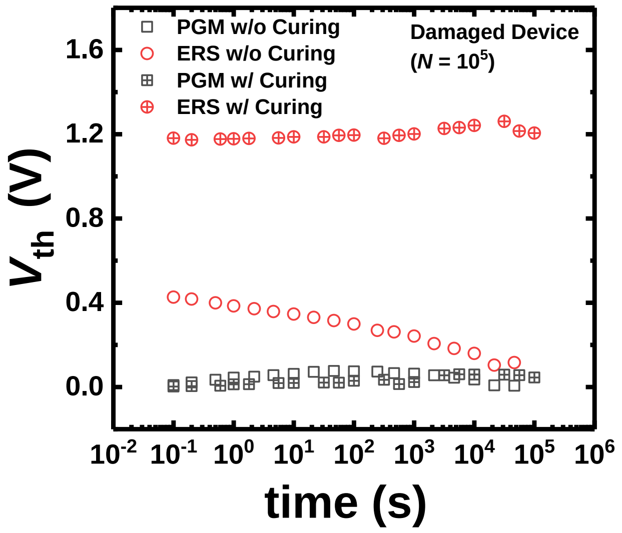


Figure S10. Threshold voltages ($V_{th}$) of ferroelectric field-effect transistors (FeFETs) at the program and erase states versus the number of program/erase (P/E) cycles. A significant improvement in retention characteristics is observed by adopting the self-curing method.

Supplementary Note 1

Mulaosmanovic et al. reported a self-curing method in FeFET [49]. However, there are major differences between [49] and this study. Firstly, the proposed self-curing methods are different. While [49] utilizes the drain (source)-body p-n junctions for the curing, this study proposed the curing method based on punch-through current. To use the drain-body junction current as the curing current, there has to be an additional body contact, which complicates the fabrication process. More importantly, even the body contact is formed, the selective curing of the device in the array is almost impossible, and curing efficiency would be significantly degraded. This is because the synaptic devices share the single body contact in the entire array. Therefore, selective curing is not possible. Furthermore, even the larger current is generated by the forward current at the p-n junction because the current flow over the entire bulk of the Si substrate, the JH will be dissipated easily, and thus the curing effects in the device would be very low. On the contrary, the proposed self-curing method using punch-through current can be applied to the selected device because the $V_{GS}$ and $V_{DS}$ can be applied to the synaptic FeFETs selectively. Moreover, the purpose of self-curing is different. While [49] demonstrated that the curing method could be used in the single memory device, this study demonstrates the efficiency of the self-curing method in the synaptic array. As shown in Fig. 5(c), the performance of the synaptic FeFETs can be significantly improved by utilizing the proposed self-curing method, and its effects on neuromorphic CNN are demonstrated for the first time in this study.
